# Single nucleotide variants in the *CCL2*, *OAS1* and *DPP9* genes and their association with the severity of COVID-19 in an Ecuadorian population

**DOI:** 10.3389/fcimb.2024.1322882

**Published:** 2024-04-17

**Authors:** Erik Chávez-Vélez, Francisco Álvarez-Nava, Alisson Torres-Vinueza, Thalía Balarezo-Díaz, Kathya Pilataxi, Camila Acosta-López, Ivonne Z. Peña, Katherin Narváez

**Affiliations:** ^1^ Carrera de Biología, Facultad de Ciencias Biológicas, Universidad Central del Ecuador, Quito, Ecuador; ^2^ Unidad de Cuidados Críticos de Adultos, Hospital Quito Sur del Instituto Ecuatoriano de Securidad Social, Quito, Ecuador

**Keywords:** association genetic study, COVID-19, *CCL2*, *DPP9*, *OAS1*, gene variant, Hardy-Weinberg equilibrium, SNV

## Abstract

COVID-19 has a broad clinical spectrum, ranging from asymptomatic-mild form to severe phenotype. The severity of COVID-19 is a complex trait influenced by various genetic and environmental factors. Ethnic differences have been observed in relation to COVID-19 severity during the pandemic. It is currently unknown whether genetic variations may contribute to the increased risk of severity observed in Latin-American individuals The aim of this study is to investigate the potential correlation between gene variants at *CCL2*, *OAS1*, and *DPP9* genes and the severity of COVID-19 in a population from Quito, Ecuador. This observational case-control study was conducted at the Carrera de Biologia from the Universidad Central del Ecuador and the Hospital Quito Sur of the Instituto Ecuatoriano de Seguridad Social (Quito-SUR-IESS), Quito, Ecuador. Genotyping for gene variants at rs1024611 (A>G), rs10774671 (A>G), and rs10406145 (G>C) of *CCL2*, *OAS1*, and *DPP9* genes was performed on 100 COVID-19 patients (43 with severe form and 57 asymptomatic-mild) using RFLP-PCR. The genotype distribution of all SNVs throughout the entire sample of 100 individuals showed Hardy Weinberg equilibrium (*P*=0.53, 0.35, and 0.4 for *CCL2*, *OAS1*, and *DPP9*, respectively). The HWE test did not find any statistically significant difference in genotype distribution between the study and control groups for any of the three SNVs. The multivariable logistic regression analysis showed that individuals with the GG of the *CCL2* rs1024611 gene variant had an increased association with the severe COVID-19 phenotype in a recessive model (*P* = 0.0003, OR = 6.43, 95% CI 2.19-18.89) and for the *OAS1* rs10774671 gene variant, the log-additive model showed a significant association with the severe phenotype of COVID-19 (*P*=0.0084, OR=3.85, 95% CI 1.33-11.12). Analysis of haplotype frequencies revealed that the coexistence of GAG at *CCL2*, *OAS1*, and *DPP9* variants, respectively, in the same individual increased the presence of the severe COVID-19 phenotype (OR=2.273, 95% CI: 1.271-4.068, *P*=0.005305). The findings of the current study suggests that the ethnic background affects the allele and genotype frequencies of genes associated with the severity of COVID-19. The experience with COVID-19 has provided an opportunity to identify an ethnicity-based approach to recognize genetically high-risk individuals in different populations for emerging diseases.

## 1 Introduction

The severe acute respiratory syndrome coronavirus 2 (SARS-CoV-2) is the pathogen responsible for the extremely contagious infection known as coronavirus disease 2019 (COVID-19). The virus has spread around the world since it first appeared in Wuhan, China, in late 2019 and reached Ecuador in February 2020 (Guevara et al., 2022). One of the countries with the most severe impact worldwide has been Ecuador. According to the Coronavirus (COVID-19) Dashboard (https://covid19.who.int/) by the World Health Organization (WHO), as of March 2023, the pandemic had accumulated more than one million confirmed cases in Ecuador, including over 36,000 deaths (WHO, 2023) with a cumulative mortality rate of 204.9 per 100,000 inhabitants (Observatorio Social del Ecuador, 2024 https://www.covid19ecuador.org/fallecidos). The COVID-19 pandemic has had profound effects on Ecuador’s health systems as well as its social, economic, and political areas. As humanity emerges from the pandemic, public health research continues to focus on understanding COVID-19 and protecting populations from its future impacts.

SARS-CoV-2 infection causes a broad clinical spectrum, ranging from asymptomatic-mild form to acute respiratory distress syndrome (ARDS) requiring invasive mechanical ventilation and potentially leading to death (Wang et al., 2020; Pairo-Castineira et al., 2021). Furthermore, there is evidence of individual differences in susceptibility to COVID-19 (Wang et al., 2020; Fricke-Galindo and Falfán-Valencia, 2021). Host-associated variables, such as advanced age, male sex, hypertension, and diabetes mellitus, have been shown to significantly influence the susceptibility and severity of COVID-19 (Biswas et al., 2021; Bollyky et al., 2022). However, these risk variables are insufficient to explain fully the great phenotypic variability of COVID-19. The COVID-19 phenotype (susceptibility to infection and/or disease severity) is believed to be a complex trait influenced by various genetic and environmental factors. Due to the unpredictable nature of the COVID-19 course, significant efforts have been made to identify reliable prognostic factors. However, the predictive capacity of such biomarkers to detect individuals at risk of developing the severe form of COVID-19 in different ethnic populations is limited. In addition, ethnic differences have been described in relation to age-standardized diagnosis rates, hospitalizations, and mortality during the pandemic (Tai et al., 2022). A complex genetic predisposition may contribute to adverse COVID-19 outcomes (Niemi et al., 2022). This is supported by findings such as family clustering of severe cases and severe disease presentation among young women with no comorbidities. It is unclear whether genetic variations contribute to the increased risk of susceptibility and severity observed in Latin-American individuals (Niedzwiedz et al., 2020). Other factors, such as high poverty levels, poor vaccination campaigns, larger multigenerational households, and a higher prevalence of comorbidities, may explain the higher rates of susceptibility and death due to SARS-CoV-2 infection in these populations (Mathur et al., 2021). Therefore, it is essential to have a more comprehensive understanding of the genetic factors that contribute to the risk of Latin-American populations. This knowledge will aid in population risk stratification and the implementation of preventive measures to protect the most vulnerable individuals.

The COVID-19 Host Genetics Initiative (https://www.covid19hg.org/), is a global network dedicated to investigating the role of human genetics in SARS-CoV-2 infection and COVID-19 severity. Chromosomal regions associated with increased disease susceptibility (*OAS1*, *OAS2*, and *OAS3* genes, a gene cluster that encodes antiviral restriction enzyme activators), pulmonary fibrosis and antigen presentation and antiviral signaling (*DPP9* gene), association with innate immune response (*IFNAR2*) and activation to cytokine storm (*TYK2*) have been reported (Pairo-Castineira et al., 2021; Velavan et al., 2021). In a meta-analysis of 46 studies from 19 countries, totaling 49,562 cases and 2 million controls, researchers found a significant association at 3p21.31. Carriers of the C allele of SNV rs10490770 of *LZTFL1* gene with an age <60 years had a significantly increased risk of progressing to death or severe respiratory failure (Genomewide Association Study of Severe Covid-19 with Respiratory Failure, 2020; Nakanishi et al., 2021). These genome-wide association studies (GWASs) are mainly conducted in developed countries with well-established health systems, better infrastructure, and qualified scientific staff. In addition, a difference in allele and genotype frequencies at SNV rs10490770 among ethnic groups was found, which correlated with susceptibility and severity for COVID-19 in individuals of South Asian ancestry (Downes et al., 2021). Variations in allele and genotype frequencies in other SNVs that confer risk for COVID-19 have also been described in populations of African descent (Barash et al., 2020). This supports the hypothesis that genetic differences could contribute to ethnic disparities in the observed COVID-19 phenotype. On the other hand, the creation of vaccines has been shown to be the most successful strategy for limiting the spread of COVID-19 (Chen et al., 2022; Nohynek and Wilder-Smith, 2022). Nevertheless, the efficacy and durability of vaccines have been questioned due to the emergence of new variants of concern (VOC) with different epidemiological and biological features (Cao et al., 2022; Chenchula et al., 2022). Therefore, obtaining more data on human genetic variants in Latin American populations is crucial due to high interbreeding. This will better prepare global health policymakers for future pandemics.

The *CCL2* gene is part of a cluster of cytokine genes on the long arm of chromosome 17. This gene encodes a protein that acts as a ligand for the C-C chemokine receptors CCR2 and CCR4 and induces a strong chemotactic response. Chemokines are a superfamily of secreted proteins that play a role in immunoregulatory and inflammatory processes. Neural tube defects and human immunodeficiency virus type 1 are among the diseases associated with the *CCL2* gene. The cytokine CCL2 is chemotactic for monocytes and basophils, but not for neutrophils or eosinophils. The migration of monocytes and macrophages to sites of infection and inflammation is strongly supported by CCL2. Its function in the early immune response is essential for the initiation of a successful antiviral defense (Conrady et al., 2013). SARS-CoV-2 is associated with increased expression of the encoded protein (Bagheri-Hosseinabadi et al., 2024). The SNV rs1024611 of the *CCL2* gene was significantly associated with COVID-19 severity (including the 1000 Genomes Project, *P* = 0.001) (Rüter et al., 2022). On the other hand, the *OAS1* gene is located at 12q24.13, a chromosomal region previously associated with the severe form of COVID-19 in genome-wide association studies (Pairo-Castineira et al., 2021). The *OAS1* gene encodes the enzyme 2′-5′ oligoadenylate synthetase 1 (2-5A), an activator of ribonuclease L (RNaseL), that degrades viral RNA in the host cell, blocks viral replication and inhibits viral protein synthesis. The SNV rs10774671 of the *OAS1* gene is a G→A transition in the last nucleotide of intron 5 of the *OAS1* gene, which affects the nonsense-mediated decay and the splicing site and controls the differential expression of isoforms with lower enzymatic activity (Bonnevie-Nielsen et al., 2005; Kjær et al., 2014). Immunodeficiency 100 with Pulmonary Alveolar Proteinosis and Hypogammaglobulinemia and Infantile-Onset Pulmonary Alveolar Proteinosis-Hypogammaglobulinemia are two diseases associated with the *OAS1* gene. In addition, SNVs in this gene have been associated with susceptibility to viral infections, including diabetes mellitus type 1 and SARS-CoV-2. Similarly, the *DDP9* gene is located on chromosomal region 19p13.1. This region was also found to be associated with disease severity in COVID-19 (Pairo-Castineira et al., 2021; Pathak et al., 2021). This gene encodes a serine protease, with post-proline dipeptidyl aminopeptidase activity that cleaves off N-terminal dipeptides from proteins having a Pro or Ala residue at position 2. DDP9 functions as a key inhibitor of caspase-1-dependent monocyte and macrophage pyroptosis in quiescent cells by delaying activation of NLRP1 and CARD8, which form the active part of the inflammasomes (Hollingsworth et al., 2021). *DPP9*-related diseases include Hatipoglu Immunodeficiency Syndrome and Interstitial Lung Disease 2. Associated pathways include pulmonary fibrosis. Suppression of pyroptosis and inflammasome activation play is now known to play a key role in breaking the cycle of viral replication and preventing amplification of SARS-CoV-2 (Sefik et al., 2022). DDP9 also plays an important role in mediating lung damage in cases of severe COVID-19 (Wang et al., 2021). Two intronic polymorphisms in the *DPP9* gene have been associated with the severe form of COVID-19 and worse clinical outcome in infected individuals (Pairo-Castineira et al., 2021; Horowitz et al., 2022).

Thus, the aim of this study was to investigate the potential association between genic variants at rs1024611 (A>G), rs10774671 (A>G), and rs10406145 (G>C) of *CCL2*, *OAS1*, and *DPP9* genes, respectively, and the severity of COVID-19 in a population from Quito, Ecuador.

## 2 Materials and methods

### 2.1 Design and study subjects

This observational case-control study was conducted at the Carrera de Biologia from the Universidad Central del Ecuador and the Hospital Quito Sur of the Instituto Ecuatoriano de Seguridad Social (Quito-SUR-IESS), Quito, Ecuador. The hospital served as a referral center for COVID-19 in Quito, Ecuador. The study included 100 patients with COVID-19 who voluntarily participated. COVID-19 was diagnosed based on clinical and radiologic findings upon hospital admission. However, the study only included patients who were confirmed to have SARS-CoV-2 through the Reverse Transcription quantitative Polymerase Chain Reaction (RT-qPCR) test from nasal swabs at the time of hospitalization. The eligible patients were then divided into two groups: the Case group, which included patients with severe COVID-19 disease, and the Control group, which included patients with asymptomatic or mild COVID-19 disease. All subjects were from the Andean (Sierra) region of Ecuador.

The study group comprised of 43 consecutively enrolled patients of both sexes who suffered from severe COVID-19 during the Omicron VOC-dominant wave from October 2021 to March 2022. Severe phenotype was defined as patients who had a) significant respiratory distress (respiratory rate >30/min); b) a chest computed tomography (CT) scan demonstrating a viral pneumonia pattern due to diffuse infiltration of both lungs greater than 50% (CORADS 6); c) the need for mechanical ventilation due to respiratory failure (PaO2/FiO2 ≤ 100mmHg (with PEEP ≥ 5cm H2O) and SpO2/FiO2 ratio <300); and d) the need for monitoring and treatment in an intensive care unit. It is important to note that all participants in the Case Group had received two doses of the SARS-CoV-2 vaccine.

The study’s control group comprised 57 healthcare workers from the same hospital who cared for COVID-19 patients admitted to the intensive care unit during the first wave of the pandemic (March-May 2020). All subjects had the asymptomatic-mild phenotype, and none of the COVID-19 patients in the control group had been vaccinated or required mechanical ventilation during the study. Asymptomatic patients were defined as those who had a positive RT-qPCR for SARS-CoV-2 with a close contact history but no clinical symptoms or signs during follow-up. Patients with a positive RT-qPCR test for COVID-19 were considered to have the mild form of the disease if they experienced the following symptoms: fever, headache, muscle/joint pain, fatigue, cough, and sore throat; respiratory rate <30/minute; SpO2 level above 93% in room air; and no evidence of pneumonia on lung CT. The individuals in the control group evolved satisfactorily without respiratory complications or the need for hospitalization. This method of subject selection was carried out to identify COVID-19-related alleles.

Exclusion criteria for both groups included consanguineous individuals, HIV-positive subjects, children, pregnant or lactating women, refugees or displaced persons, and persons with little or no knowledge of the Spanish language.

Participants were asked to provide demographic and anthropometric data, including age, sex, height, and weight, as well as clinical information such as previous comorbidities, medication intake, and hospitalization, including intensive care unit admission. Data from asymptomatic-mild COVID-19 patients (control group) was collected using a questionnaire application by qualified medical specialists. Comorbidities reported by participants were grouped together and their combined effect was weighted in the regression analysis.

### 2.2 Ethical considerations

The study was conducted in accordance with human rights and the principles of the Declaration of Helsinki, including subsequent revisions. The research protocol (MSP-CGDES-2020-0244-O1) was approved by the Comite de Etica para la Revisión Expedita de Investigaciones en COVID-19 of the Ministerio de Salud Publica of Ecuador in accordance with relevant guidelines and regulations. Written informed consent was obtained from all participants, including healthy individuals, prior to their inclusion in the study. If participants were unable to sign due to their condition, we obtained consent from their legal representatives. Participants provided written consent for sample withdrawal, biological sample processing, and the use of medical records. The samples and data were collected by hospital staff members who had no interaction with the researchers conducting the molecular tests.

### 2.3 Molecular analysis

#### 2.3.1 DNA extraction and quality

Ten milliliters of venous blood were collected from each participant using EDTA anticoagulant tubes. DNA was extracted using the Column-Pure Blood Genomic DNA (ABM, Vancouver, Canada) kit according to the manufacturer’s instructions. The extracted DNA was quantified using the Qubit dsDNA BR ASSAY Kit (21000 ng 100RX) with the Qubit fluorometer (Invitrogen, Massachusetts, USA). The quality of the DNA was assessed using the Microtek Bio-1000F program scanner (Microtek International Inc., Hsinchu City, Taiwan) after 1.5% agarose gel electrophoresis at 80V for one hour. The isolated DNA was then diluted to a working concentration of 50 ng/μL before genotyping and stored at -20°C.

#### 2.3.2 Genotyping of single-nucleotide variants of *CCL2*, *OAS1* and *DPP9* genes

The polymerase chain reaction-based restriction fragment length polymorphism method (PCR-RFLP) was used to analyze three polymorphic SNVs: *CCL2* rs1024611, *OAS1* rs10774671, and *DPP9* rs10406145 (see **Supplementary Table S1**). The primer sequences, annealing temperature, restriction enzymes, and restriction digestion pattern are presented in **Supplementary Table S2**.

The PCR master mix and thermocycler settings (Applied Biosystems MiniAmp, Thermo Fisher Scientific Inc., Massachusetts, USA) were performed according to previously published protocols (Qiu et al., 2008; Noguchi et al., 2013; Tu et al., 2015) with modifications. The components and concentrations of each PCR run are described in detail in **Supplementary Table S3**. The PCR products were electrophoresed on 1.5% agarose gels in the blueGelTM system (48V, 45 minutes) (MiniPCR Bio, Massachusetts, USA). The products were then digested with their respective restriction enzymes and electrophoresed in 3% agarose gels using Thermo ScientificTM equipment (120V, 2 hours) (Thermo Fisher Scientific Inc., Massachusetts, USA).

PCR product sizes were used to determine the alleles and genotypes for each sample. The G allele is represented by two fragments of 91 plus 45 bp for the *CCL2* rs1024611 variants, while the A allele is indicated by a band of 136 bp. A heterozygous individual (GA) is designated by the combination of both G and A bands, i.e., 136/91/45 bp (Tu et al., 2015). The *OAS1* rs10774671 gene variant was analyzed by digesting the 306 bp PCR products with the restriction enzyme *Alu*I. The presence of the A allele was indicated by two bands, 255 and 51 bp, while the G allele was represented by a single band of 306 bp. A heterozygous genotype (GA) was demonstrated using two segments of 306 and 255 bp (Noguchi et al., 2013). The PCR products for the *DPP9* rs10406145 variant were analyzed, revealing segments of 139 and 114 bp that identified the G and C alleles, respectively. A heterozygous patient (GC) can be identified by the presence of two bands (139 and 144 bp) (Qiu et al., 2008). To ensure the reproducibility and quality of genotyping of PCR-RFLP, 10% of all samples were randomly sequenced, and the results showed complete matching.

### 2.4 Statistical analysis

The data analysis was performed using Microsoft Excel 2019, GraphPad InStat 7.00 (GraphPad Software, Inc., La Jolla, CA, USA), SNPStats (https://www.snpstats.net; RRID : SCR_002142), the HardyWeinberg statistical package for R Studio (Graffelman, 2015), the TernaryPlot webpage (https://www.ternaryplot.com/) and SHEsis software (http://analysis.bio-x.cn/; RRID : SCR_002958). The general features of the participants are described as number (n) and proportions of cases and continuous variables are expressed as mean ± standard deviation (SD) under conditions of normal distribution and skewed data as median and rank. Age was presented as both a continuous and a categorical variable (<50 and ≥50 years). The Kolmogorov–Smirnov test was used to determine the probability distributions of variables. To compare the proportions of categorical variables, i.e., differences in the percentages of sex, obesity, comorbidities, and subjects between case and control groups, the two-proportion *z*-test was used. A *Student t*-test was performed to compare means between groups for continuous variables. The association between qualitative variables and COVID-19 phenotype was examined using Chi-square (χ^2^). Results as determined by 95% confidence intervals (95% CI) and a *P*-value of <0.05 were considered to be statistically significant. The analysis considered several independent variables, including age, sex, obesity, and comorbidities, as well as alleles and genotypes. These variables were exported to a multivariable logistic regression analysis, with the severe phenotype of COVID-19 serving as the dependent variable. The purpose was to assess the individual effect of each variable on the COVID-19 phenotype and control for possible confounders. The final multivariable model set a significance level of *P* < 0.05 for two-sided tests.

Allele and genotype frequencies were calculated by direct counting and expressed as proportions and percentages. Using the exact, χ^2^, the likelihood ratio, and the permutation tests, allele and genotype frequencies were examined to determine whether the groups were in Hardy-Weinberg equilibrium (HWE) (Graffelman and Weir, 2016). A De Finetti diagram was generated using the interface from TernaryPlot webpage (https://www.ternaryplot.com/) to represent HWE. Fisher’s exact test was used to compare allele and genotype frequencies between the case and control groups. The P-values were adjusted using the Bonferroni correction to determine the significance of multiple testing at each feature genetic model (P < 0.05/n, where n is the number of alleles or genotypes analyzed).

The odds ratios along with the corresponding 95% CI between the wild-type and the mutant alleles were tested for each SNV to examine the strength of associations between the different alleles and clinical phenotype of COVID-19. The association between SNVs and the existence of a severe phenotype in COVID-19 was then further examined in different genetic models (crude analysis). Adjusted odds ratios were used to assess the association between *CCL2*, *OAS1*, and *DPP9* genotypes and the presence of severe COVID-19 phenotype, after controlling for age, sex, obesity, and comorbidities.

Linkage disequilibrium (LD) and haplotype blocks were analyzed among the three SNVs using SHEsis freeware (online version). The degree of LD was evaluated using the standardization coefficient (*D′*) and the correlation coefficient (*r^2^*). The stronger LD between two SNVs was defined as having values of *D’* or *r^2^* that were closer to one.

## 3 Results

### 3.1 Demographic characteristics of participants

The clinical characteristics of the subjects in this study are described in **Table 1**. A total of 100 patients with COVID-19 with a mean ± SD of 49.47 ± 12.59 years old (median (P10, P90)/(min, max) of 49.21 (32.13, 67.38)/(25.25, 80.08) years old), including 63 males and 37 females, were enrolled from subjects admitted to Quito-SUR-IESS Hospital from Quito, Ecuador. Fifty-seven COVID-19 patients with an asymptomatic-mild phenotype from healthy staff served as the control group and 43 patients with severe COVID-19 served as the case group. Comorbidities were reported in 14 participants (study group (n=10) and control group (n=4)), including arterial hypertension (n=6), diabetes mellitus type I (n=1) and type 2 (n=4), rheumatoid arthritis (n=2), epilepsy (n=1); chronic renal insufficiency (n=1) and chronic obstructive pulmonary disease (n=1). Four patients had more than one comorbidity. A male preponderance was observed in the case group (male vs female: 33 vs 10), whereas there was no sex difference in the male to female ratio in the control group (male to female: 30-to-27). As a result, a statistically significant sex difference was found between the case and control groups (P= 0.013). Patients with the severe phenotype of COVID-19 were also older (P= 0.0003) and more likely to be obese (P= 0.0030) than those with the asymptomatic-mild phenotype. Additionally, individuals in the case group were more likely to have comorbid conditions than individuals in the control group (P=0.0203).

**Table 1:** Clinical features of participants.

Clinical Features		Case Group^†^ (n=43)		Control Group^†^ (n=57)		*z*/χ^2^	*P*
		n	Proportion	n	Proportion (%)		
Age (years)	Mean ± SD	56.77 ± 12.11		43.97 ± 9.93		4.66	<0.0001*
	< 50	14	0.33	39	0.68	12.655	0.0003
	≥ 50	29	0.67	18	0.32		
Sex	Male	33	0.77	30	0.53	6.113	0.0134
	Female	10	0.23	27	0.47		
Obesity		15	0.35	6	0.11	2.960	0.0030
Comorbidities		10	0.23	4	0.07	2.316	0.0203

† Subjects from the control group were unvaccinated, whereas all individuals in the case group had received two doses of the SARS-CoV-2 vaccine. * Paired t-test. Kolmogorov–Smirnov test statistic is 0.08062.

### 3.2 Comparison of allele and genotype frequencies of SNVs in *CCL2* rs1024611, *OAS1* rs10774671 and *DPP9* rs10406145 between case and control groups and Hardy-Weinberg test

The allele and genotype frequencies for each SNV in the *CCL2*, *OAS1*, and *DPP9* genes are listed in **Table 2**. The results showed that the minor allele frequencies (MAFs) of the *OAS1* rs10774671 and *DPP9* rs10406145 gene variants were low, ranging from 0.16 to 0.19 in the case group and 0.12 to 0.21 in the control group, respectively. In comparison, the MAFs for the *CCL2* rs1024611 gene variant were higher in the case and control groups (0.23 and 0.49, respectively). The allele frequencies of *OAS1* and *DPP9* showed no significant difference between the case and control groups (*z*-statistic and *P*-value: 0.428 and 0.669, and 0.804 and 0.421, respectively). The frequency of the *CCL2* rs1024611-G allele was significantly higher in the case group (OR = 3.186, 95% CI 1.713 to 5.963, *z*-statistic: 3.66 and *P*=0.0003).

**Table 2:** Allele and Genotype Frequencies and Hardy-Weinberg equilibrium in SNVs.

SNV allele frequencies (n=100)							
Allele	All subjects		Case Group		Control Group		χ^2^/Fisher
	Count	Proportion	Count	Proportion	Count	Proportion	*P*
CCL2							
G	124	0.62	66	0.77	58	0.51	0.0001
A	76	0.38	20	0.23	56	0.49	
OAS1							
A	160	0.8	70	0.81	90	0.79	0.668
G	40	0.2	16	0.19	24	0.21	
DPP9							
G	172	0.86	72	0.84	100	0.88	0.419
C	28	0.14	14	0.16	14	0.12	
SNV genotype frequencies (n=100)							
Genotype	All subjects		Case Group		Control Group		
CCL2							
GG	40	0.4	27	0.63	13	0.23	
GA	44	0.44	12	0.28	32	0.56	
AA	16	0.16	4	0.09	12	0.21	
OAS1							
GG	2	0.02	0	0	2	0.04	
AG	36	0.36	16	0.37	20	0.35	
AA	62	0.62	27	0.63	35	0.61	
DPP9							
GG	75	0.75	30	0.7	45	0.79	
GC	22	0.22	12	0.28	10	0.18	
CC	3	0.03	1	0.02	2	0.04	
Exact test for Hardy-Weinberg Equilibrium							
***CCL2***		**GG**	**AA**	**GA**	**G**	**A**	***P*-value**
All subjects		40	44	16	124	76	0.53
Case Group		27	12	4	66	20	0.19
Control Group		13	32	12	58	56	0.43
***OAS1***		**GG**	**AA**	**AG**	**G**	**A**	
All subjects		62	36	2	160	40	0.35
Case Group		27	16	0	70	16	0.31
Control Group		35	20	2	90	24	1
***DPP9***		**GG**	**CC**	**GC**	**G**	**C**	
All subjects		75	22	3	172	28	0.4
Case Group		30	12	1	72	14	1
Control Group		45	10	2	100	14	0.18

**Figure 1:**
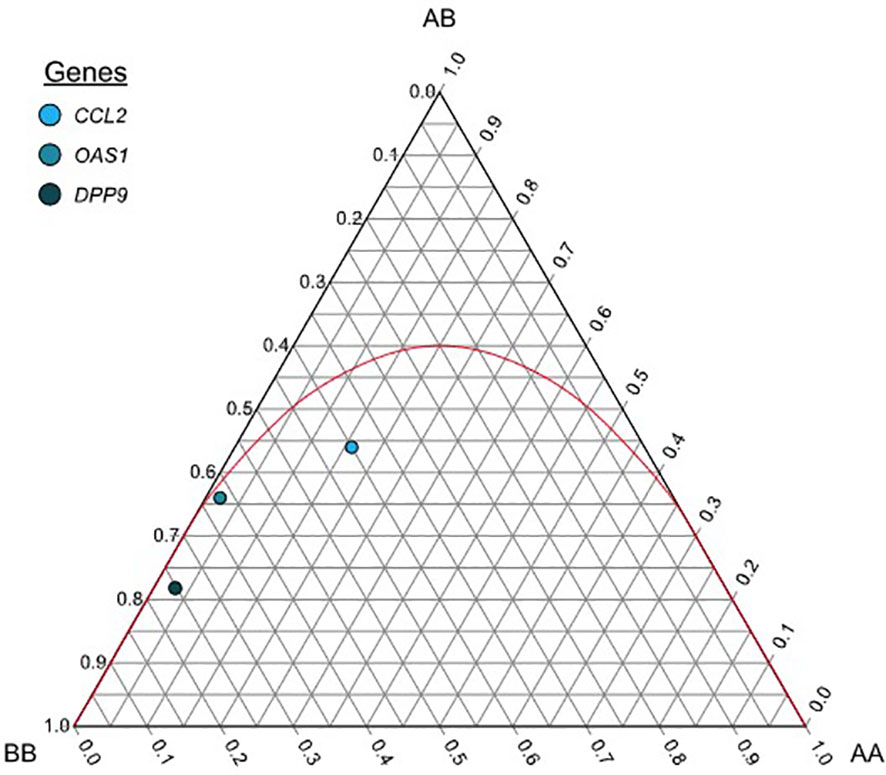
De Finetti diagram each dot shows allele and genotype frequencies for the general group of the following markers: rs1024611 (A>G) for CCL2, rs10774671 (A>G) for OAS1, and rs10406145 (G>C) for DPP9. The parable in red shows the acceptance region for the Hardy-Weinberg equilibrium, markers that fall below the parable are said to be in HWE. AA= Homozygous for the major allele, AB= Heterozygous, BB=Homozygous for the minor allele.

Hardy-Weinberg equilibrium was observed in the genotype distribution of all SNVs in the entire sample of 100 individuals (exact test; *P*=0.53, 0.35, and 0.4 for *CCL2*, *OAS1*, and *DPP9*, respectively) (**Table 2**, **Supplementary Table S4**, and **Figure 1**). In addition, the HWE test revealed no statistically significant difference in the genotype distribution between the study and control groups for any of the three SNVs.

### 3.3 Univariate analysis of the associations between the alleles and genotypes of three loci and the presence of the severe phenotype of COVID-19

For each of the three SNVs considered, we examined the odds ratios between the wild-type and the mutant alleles. The association between the SNVs and the presence of a severe phenotype in COVID-19 was then further investigated in different genetic models.

No allele or genotype of the *OAS1* or *DPP9* loci was significantly associated with the severe COVID-19 phenotype. However, in a recessive model, the GG genotype of the *CCL2* rs1024611 gene variant showed a significant association with the severe clinical manifestation of COVID-19 (*P*<0.0001, OR = 5.71, 95% CI 2.38-13.70), while in an overdominant model, the AA genotype showed a protective effect against the severe clinical phenotype of (*P*=0.0044, OR = 0.30, 95% CI 0.13-0.71).

### 3.4 Multivariable logistic regression analysis of the associations between the alleles and genotypes of three loci and the presence of the severe phenotype of COVID-19

The real association between the *CCL2*, *OAS1*, and *DPP9* variants and the presence of the severe COVID-19 phenotype was examined using multivariable logistic regression analysis to control the influence of age, sex, obesity, and comorbidities. Similarly, none of the alleles or genotypes of the *DPP9* gene variant were significantly associated with the severe phenotype of COVID-19 according to the results of the multivariable logistic regression analysis (**Table 3**). When the GG genotype of the *CCL2* rs1024611 gene variant was examined among individuals with different clinical manifestations of COVID-19, its distribution was associated with an increased association with the severe COVID-19 phenotype in a recessive model (*P* = 0.0003, OR = 6.43, 95% CI 2.19-18.89). Similarly, the AG genotype showed a protective effect against the severe form of COVID-19 in an over dominant model (*P*=0.0029, OR = 0.21, 95% CI 0.07-0.62). For the *OAS1* rs10774671 gene variant, the log-additive model showed a significant association with the severe phenotype of COVID-19 (*P*=0.0084, OR=3.85, 95% CI 1.33-11.12).

**Table 3:** SNV associations with the severe form of COVID-19 (n=100, adjusted analysis by covariables).

CCL2							
Model	Genotype	Control Group	Case Group	OR (95% CI)	P-value	AIC	BIC
**Codominant**	AA	12 (21.1%)	4 (9.3%)	1.00	**0.0014**	107.1	125.3
	AG	32 (56.1%)	12 (27.9%)	0.67 (0.14-3.29)			
	GG	13 (22.8%)	27 (62.8%)	4.81 (1.01-22.89)			
**Dominant**	AA	12 (21.1%)	4 (9.3%)	1.00	0.39	117.5	133.1
	AG-GG	45 (79%)	39 (90.7%)	1.84 (0.44-7.70)			
**Recessive**	AA-AG	44 (72.2%)	16 (37.2%)	1.00	**0.0003**	105.3	121
	GG	13 (22.8%)	27 (62.8%)	**6.43 (2.19-18.89)**			
**Overdominant**	AA-GG	25 (43.9%)	31 (72.1%)	1.00	**0.0029**	109.3	125
	AG	32 (56.1%)	12 (27.9%)	**0.21 (0.07-0.62)**			
**Log-additive**	---	---	---	3.09 (1.41-6.79)	0.0027	109.2	124.8
OAS1							
**Codominant**	GG	2 (3.5%)	0 (0%)	1.00	0.0047	109.5	127.7
	AG	20 (35.1%)	16 (37.2%)	0.00 (0.00-NA)			
	AA	35 (61.4%)	27 (62.8%)	0.00 (0.00-NA)			
**Dominant**	GG	2 (3.5%)	0 (0%)	1.00	0.0046	110.2	125.8
	AG-AA	55 (96.5%)	43 (100%)	0.00 (0.00-NA)			
**Recessive**	GG-AG	22 (38.6%)	16 (37.2%)	1.00	0.045	114.2	129.8
	AA	35 (61.4%)	27 (62.8%)	3.00 (0.98-9.21)			
**Overdominant**	GG-AA	37 (64.9%)	27 (62.8%)	1.00	0.25	116.9	132.5
	AG	20 (35.1%)	16 (37.2%)	0.54 (0.19-1.56)			
**Log-additive**	---	---	---	**3.85 (1.33-11.12)**	**0.0084**	111.3	126.9
DPP9							
**Codominant**	CC	1 (2.3%)	2 (3.5%)	1.00	0.17	116.7	134.9
	GC	12 (27.9%)	10 (17.5%)	0.10 (0.00-2.12)			
	GG	30 (69.8%	45 (79%)	0.25 (0.01-4.40)			
**Dominant**	CC	1 (2.3%)	2 (3.5%)	1.00	0.29	117.1	132.7
	GC-GG	42 (97.7%)	55 (96.5%)	0.23 (0.01-3.94)			
**Recessive**	CC-GC	13 (30.2%)	12 (21.1%)	1.00	0.28	117.1	132.7
	GG	30 (69.8%)	45 (79%)	1.81 (0.61-5.37)			
**Overdominant**	CC-GG	31 (72.1%)	47 (82.5%)	1.00	0.11	115.6	131.3
	GC	12 27.9%	10 17.5%	0.39 (0.12-1.25)			
**Log-additive**	---	---	---	1.28 (0.52-3.16)	0.59	117.9	133.6

Covariables: age (<50 and ≥50 years), sex, obesity, and comorbidities. Bold font represents OR (95% CI) and P values which are statistically significant.

### 3.5 Stratification analysis of the correlation between *CCL2*, *OAS1*, and *DPP9* variants and COVID-19

Using age-based stratification analysis in adjusted genetic models, the interaction of *CCL2*, *OAS1*, and *DPP9* SNVs with the presence of the severe COVID-19 phenotype was examined. No interaction between age and *CCL2* rs1024611 and *OAS1* rs1024611 variants was associated with severe COVID-19. In contrast, the CC genotype at *DPP9* rs10406145 was associated with severe COVID-19 in individuals younger than 50 years in an adjusted dominant model (interaction P value=0.021, OR=7.67, 95% CI 2.75-21.42). On the other hand, a significant association between the GG genotype of the *DPP9* rs10406145 gene variant and the severe form of COVID-19 was found in males in an adjusted recessive genetic model (OR = 4.47, 95% CI 1.04-19.21).

### 3.6 Analysis of *CCL2*, *OAS1* and *DPP9* SNV haplotypes and correlation with severe phenotype of COVID-19

For rs1024611 in *CCL2*, rs10774671 in *OAS1*, and rs10406145 in *DPP9* gene variants, further haplotype block analysis and linkage disequilibrium were performed (**Supplementary Table S5**). Haplotype block analysis revealed that two of the three loci formed a single haplotype block. However, the haplotypes generated from this block were not associated with the severe COVID-19 phenotype (**Table 4**). Frequency distributions of seven major haplotypes (AAC, AAG, AGG, GAC, GAG, GGG, AGC, and GGC, identified by SHEsis software) in cases and controls are shown in **Table 5**. When the ACC, AAG, and GAG haplotype frequencies were compared between individuals with severe COVID-19 and asymptomatic-mild controls, the results indicated a significant difference between the frequencies of these haplotypes in patients with severe COVID-19 (*P*=0.001598). Haplotype frequency analysis revealed that the coexistence of *CCL2* rs1024611-G, *OAS1* rs10774671-A, and *DPP9* rs10406145-G alleles in the same individual increased the presence of the severe COVID-19 phenotype (OR=2.273, 95% CI: 1.271-4.068, P=0.005305). In contrast, the frequency of the AAG haplotype was significantly lower in patients with the severe COVID-19 phenotype than in controls (*P*=0.000804), which was associated with a protective effect against the severe clinical COVID-19 phenotype (OR = 0.328, 95% CI: 0.168-0.639). Other haplotype frequencies and the COVID-19 phenotype did not significantly correlate.

**Table 4:** Haplotype Distribution of *OAS1* rs10774671 and *DPPL6* rs10406145 gene variants in the Case and Controls Groups.

Haplotype	Cases (n/%)	Control (n/%)	χ^2^	*P*	OR (95% CI)
AG	19	26	0.0143	0.9047	1.888 (0.886-4.022)
AC	7	9	0.004	0.9496	1.037 (0.368-2.916)
GG	11	19	0.6818	0.4089	0.667 (0.296-1.502)
GC	5	1	------	0.0539*	7.368 (0.844-64.32)

*Fisher´s test applied: actual value less than 5.

**Table 5:** Haplotype analysis of the loci *CCL2*, *OAS1*, and *DPP9*.

	Case (freq)	Control (freq)	χ^2^	Fisher´s *P*	Pearson`s *P*	Odds Ratio [95%CIJ
A A C	0.00 (0.000)	6.29 (0.055)	4.898	0.026925	0.026902	-----
**A A G**	15.41 (0.179)	45.56 (0.400)	11.245	**0.000804**	**0.000803**	**0.328 (0.168-0.639)**
A G G	4.47 (0.052)	4.15 (0.036)	0.287	0.592180	0.592179	1.450 (0.370—5.688)
G A C	11.94 (0.139)	7.71 (0.068)	2.802	0.094211	0.094149	2.222 (0.857—5.761)
**G A G**	42.65 (0.496)	34.44 (0.302)	7.779	**0.005305**	**0.005299**	**2.273 (1.271-4.068)**
G G G	9.47 (0.110)	15.85 (0.139)	0.371	0.542605	0.542598	0.766 (0.325—1.808)
A G C	0.12 (0.001)	0.00 (0.000)	0.165	0.684247	0.684248	-----
G G C	1.94 (0.023)	0.00 (0.000)	2.595	0.107269	0.107202	-----

Global result: Global χ^2^ = 23.202652 (df—7; frequency <0.001 in both case and control group has been dropped). Fisher´s P-value is 0.001598 and Pearson´s P value is 0.001571. Bold font represents P values and OR (95% CI) which are statistically significant.

## 4 Discussion

Genome-wide association studies (GWAS) and candidate SNV association analyses have identified genes associated with the COVID-19 phenotype (Pairo-Castineira et al., 2021; Pathak et al., 2021; Rüter et al., 2022). Numerous studies indicate that host genetic variants play a significant role in the severity of COVID-19 (Williams et al., 2020); as a result, the interaction of genetic and environmental factors may partially account for the observed phenotypic spectrum in COVID-19. Furthermore, there are data highlighting ethnic differences in the prevalence of the severe phenotype of COVID-19 (Webb Hooper et al., 2020; Oda et al., 2021; Ishak et al., 2022; Tai et al., 2022). Because different populations have distinct genetic backgrounds, there may be variations in the allele and genotype frequencies of genes associated with COVID-19. Consequently, the role of these gene variants may vary among ethnically different populations. Several candidate genes have been evaluated to estimate their association with COVID-19, but the effect of gene variants on the phenotypic spectrum of COVID-19 in Latin American populations is largely unexplored and needs to be investigated. The present study focused on the association between the SNVs *CCL2* rs1024611, *OAS1* rs10774671, and *DPP9* rs10406145 and severe COVID-19 in a population from Quito, Ecuador. We compared the allele and genotype frequencies in an attempt to find genetic markers that are associated with the COVID-19 phenotype in a mixed Ecuadorian population with a high level of Native American ancestry. The results of the current study support the notion that the geographic location of populations affects the allele and genotype frequencies of SNVs, and they also emphasize the importance of genetic population studies in ethnically diverse groups.

Ecuador is currently a multiethnic society. Multiple migration and admixture events, including Native American settlements and the movement of Western European and Sub-Saharan African individuals, have influenced on the complex pre- and post-Columbian demographic history of Ecuador. However, patterns of admixture varied widely across Ecuador, in part due to geographic region (Santangelo et al., 2017; Nagar et al., 2021). For example, Mestizos (mixed individuals) are the most prevalent ethnic group in the Andean highlands (Sierra) of Ecuador, although Native American ancestry is estimated to make up about 65% of this Mestizo population, which is higher than any other mixed American group observed in South America, except for Peru (Santangelo et al., 2017; Nagar et al., 2021).

For the gene variants analyzed in this study, few publications have reported allele and genotype frequencies for Latin American populations. The MAF for the rs1024611 A→G variant of the *CCL2* gene was 0.28 in 233,286 individuals, according to data from the Allele Frequency Aggregator (ALFA) pipeline of the National Center for Biotechnology Information (NCBI). This contrasts with the results of the present study. In the Asian population, the G allele of this gene variant is 0.56, which is closer to our study. However, the frequency reported in groups in Mexico and Peru with a strong Native American ancestry is comparable to that found in the current study (Flores-Villanueva et al., 2005; Ganachari et al., 2012). Notably, the frequency of the A allele for the rs1024611 variant of the *CCL2* gene was higher in a Latin American population with a high Western European ancestry compared to that found in our study (Velez Edwards et al., 2012).

The same scenario is also observed for rs10774671 of *OAS1* and rs10406145 of *DPP9* gene variants in our study, where the allele and genotype frequencies are very similar in Latin American populations with high Native American ancestry (Sánchez-González et al., 2021) and with East Asian populations, but they differ significantly from Western European and African populations (European Bioinformatics Institute, 2023). The correspondence in the genetic structure between the East Asian population and the participants in our study could be explained by a common ancestral origin, as has been reported for the HLA marker system between Han Chinese and South American Native populations (Tokunaga et al., 2001). Native Americans are descended from migrations of Asian populations that crossed the Bering land bridge from East Asia during glacial periods 15,000 to 30,000 years ago. However, the fixation of alleles in populations with recent admixture, such as the Ecuadorian population, may be influenced by other factors such as genetic drift, including bottleneck and founder effects. This similarity also seems to be observed in the clinical setting. For example, in a German study, the G allele in the rs1024611 variant of *CCL2* was found to have a protective effect against the severe form of COVID-19 in the codominant model (OR = 0.56, 95% CI = 0.39-0.78; *P* = 0.001), and the frequency of the A allele was higher than that reported in our study (Rüter et al., 2022).

In contrast, our data showed that the GG genotype is associated with the severe COVID-19 phenotype in a recessive model. The GG genotype was also consistently associated with SARS-CoV infection in four independent case-control studies in populations of Chinese ancestry (Tu et al., 2015). Thus, the clinical effect of these SNVs may vary in ethnically diverse populations. In a molecular context, the gene variant has been shown to confer increased transcriptional activity with higher levels of CCL2 mRNA and protein *in vivo* and *in vitro*. In addition, greater leukocyte tissue infiltration has been reported in carriers of the GG genotype compared to individuals with the A allele (Gonzalez et al., 2002; Mühlbauer et al., 2003; McDermott et al., 2005; Li et al., 2022). Thus, individuals with the GG genotype may have a higher expression of *CCL2* and, consequently, a higher attraction of monocytes and macrophages, which is associated with the severe COVID-19 phenotype.

Our study showed a significant increase in the frequency of the G allele (77%) and the GG genotype (63%) in *CCL2* in individuals in the case group compared to those in the control group. These findings suggest a possible association between the rs1024611-G variant in *CCL2* and the severe form of COVID-19 in an Ecuadorian population. In addition, we found that the *CCL2* rs1024611-G allele had a significantly higher frequency in the case group after adjustment for age (<50 and ≥50 years), sex, obesity (BMI ≥30), and comorbidities, and rs1024611-G was found to be associated with the severe phenotype of COVID-19 in a recessive genetic model. Similarly, the *OAS1* rs1024611-GG genotype was found to be associated with the severe COVID-19 phenotype in a log-additive genetic model. In contrast, no significant associations with the severe COVID-19 phenotype were found for the allele or genotype of *DPP9* rs10406145 in our study. However, we showed that the *DPP9* rs10406145-GG genotype was more common in the asymptomatic-mild group compared to individuals with the severe COVID-19 phenotype. Nonetheless, this latter difference did not meet the more stringent 0.005 level of statistical significance required by the Bonferroni correction. This association would likely reach significance in a study with a larger cohort.

Although the genes analyzed in our study have been associated with monogenic and multifactorial traits (coronary artery disease, immunodeficiency 100 with pulmonary alveolar proteinosis and hypogammaglobulinemia, resistance to HIV-1, susceptibility to Mycobacterium tuberculosis and spina bifida, among others), no correlation was found between *CCL2* and any COVID-19-related comorbidities in a GWAS that included inflammatory protein-coding genes in older males (Hillary et al., 2020). Furthermore, another study that used the GWAS approach to relate changes in metabolite and protein levels, including *CCL2*, to intermediate phenotypes found no correlation with COVID-19-related comorbidities (Suhre et al., 2017). However, in one study, the *OAS1* gene rs11066453 variant (p=4.53×10-9) was associated with 1-hour oral glucose tolerance test (OGTT) hyperglycemia (1-hPG), but not fasting plasma glucose or 2-hP (Go et al., 2013). Similarly, SNV rs10410207 in the *DPP9* gene was shown to be significantly correlated with 2-hydroxyoctanoate levels in a study assessing lipid concentrations in elite athletes (Al-Khelaifi et al., 2019). Mendelian randomization studies between genetic variations associated with COVID-19 and its comorbidities may provide relevant data in this regard.

Studies have shown that the prevalence of the severe form of COVID-19 is significantly higher in males than in females when adjusted for age and comorbidities (Williamson et al., 2020; Gao et al., 2021; Shelton et al., 2021; Song et al., 2021). In our study, individuals with the severe phenotype of COVID-19 had significantly different mean age and BMI, as well as the presence of obesity and comorbid conditions, compared with the control group. We found a significant sex difference between the case and control groups (*P*= 0.013), but the stratified analysis showed no interaction between sex and the *CCL2* rs1024611 or *OAS1* rs1024611 variants and the severe COVID-19 phenotype. However, the presence of the *DPP9* rs10406145-GG genotype showed a fourfold greater association with the severe form of COVID-19 in males than in females in a recessive genetic model (OR = 4.47, 95% CI 1.04-19.21), after adjustment for other confounding variables. In contrast, we found that in those individuals younger than 50 years, the CC genotype at *DPP9* rs10406145 was associated with severe COVID-19. Nevertheless, after Bonferroni correction, this association was not significantly associated with the severe phenotype. It is plausible that specific genes influencing the severe COVID-19 phenotype may be associated with comorbidities. However, no significant interaction was found between the gene variants analyzed and pre-existing medical comorbidities in our subjects.

Allele arrangements that tend to be inherited together are called haplotypes. Two of the three loci were found to form a single haplotype block in the current analysis. However, no association with the severe phenotype of COVID-19 was observed in the haplotypes generated from this block. According to multivariate logistic regression analysis, the *CCL2* rs1024611-GG genotype may be independently associated with severe COVID-19. However, haplotypes are often more predictive of disease-related genes than are individual SNVs. Our results showed that the severe COVID-19 phenotype was associated with the co-occurrence of the *CCL2* rs1024611-G, *OAS1* rs1024611-A, and *DPP9* rs10406145-G alleles. Thus, it is possible that the GAG haplotype may be involved in the presence of the severe phenotype of COVID-19 in the Ecuadorian population. In contrast, the frequency of the AAG haplotype, which is formed by the same genetic variants, was lower in patients with severe COVID-19 than in patients with asymptomatic-mild COVID-19. Thus, the AAG haplotype may have a protective effect against the severe phenotype of COVID-19 in the Ecuadorian population.

Our study has several strengths. To obtain a more precise effect size, we used a covariate-adjusted multivariate model associated with the presence of COVID-19 severity. We were unable to include all factors associated with COVID-19 severity. Nonetheless, we consider that we have accounted for the most important confounding variables in our study, which would make our results more robust. We also analyzed different genetic models and selected the best one based on the lowest of the AIC values and *P*-values among the four inheritance patterns, although the most ideal model has not been established (Horita and Kaneko, 2015). We also performed haplotype analysis of the alleles of non-linked genes. Thus, these gene variants are subject to the Mendelian law of independent assortment. They are not affected by the process of crossing over. Therefore, the combination of haplotypes is useful in gene-disease association analysis to more precisely associate alleles or genotypes.

The SARS-CoV-2 Omicron VOC (B.1.1.529 lineage) first emerged in South Africa in November 2021 and has since spread quickly around the world (Jassat et al., 2022). The emergence of this fifth VOC coincided with a global increase in vaccine immunity. Compared to wild type and previous VOCs, Omicron is distinguished by a number of spike protein mutations that are known to result in increased transmissibility, viral binding affinity, and antibody escape (Harvey et al., 2021; Karim and Karim, 2021; Sheikh et al., 2022). However, current evidence suggests that Omicron may be associated with a less severe clinical presentation compared with the previous variants of SARS-CoV-2 (Jassat et al., 2022). Studies from South Africa, the United Kingdom, and the United States have reported significantly lower odds of hospitalization and severe disease in Omicron-infected individuals compared to those infected with previous VOCs. According to one study, although more SARS-CoV-2 cases were documented during the Omicron wave, hospitalization rates were lower, and patients who were admitted had a much shorter median length of stay and were less likely to be severely ill than patients admitted during previous waves (Jassat et al., 2022). According to the UK Health Security Agency (UKHSA), Omicron patients were also less likely to visit or be admitted to the hospital than those infected with the Delta variant (Wolter et al., 2022). Similarly, Omicron-infected individuals were reported to have a reduced risk of hospitalization compared to Delta-infected individuals (Sheikh et al., 2022). Likewise, Omicron subjects were reported to require less respiratory support than in previous VOCs (Christensen et al., 2022). As noted above, several factors, including VOCs, reinfection with SARS-CoV-2, and vaccination, influence the clinical severity of COVID-19. Omicron has a significant reinfection rate (Pulliam et al., 2022). Omicron was reported to have a ten-fold higher potential reinfection rate than Delta VOC (Sheikh et al., 2022). The UKHSA also reported that Omicron individuals who received 2 or 3 doses of any vaccine had a lower hospitalization rate than those unvaccinated Omicron-infected subjects (Wolter et al., 2022). Furthermore, those who received the third or booster dose of the vaccine were less likely to develop symptoms following an Omicron infection. A less virulent virus and high immunity from previous infection(s) or vaccination, particularly in vaccinated individuals with re-infection (“hybrid immunity”), may have contributed to lower hospitalization rates and less severe infections among SARS-CoV-2-infected individuals during the Omicron-dominated fourth wave (Cele et al., 2022). Moreover, Omicron VOC infected bronchial cells more rapidly and alveolar cells more slowly than Delta VOC in an ex vivo study (Hui et al., 2022). This may explain the less severe infections observed in Omicron cases. Omicron has also been shown to have reduced virulence in animal models. Mice infected with Omicron had less severe disease than those treated with other variants (Halfmann et al., 2022).

Several limitations of this study must be considered. The reduced sample size and the lack of a replication-independent cohort to verify the associations were the main limitations of our study. The smaller sample size of participants in our study also limits the statistical interpretation of the significant (type I error (false positive)) and nonsignificant (type II error (false negative)) associations. In addition, because we tested genetic variants in individuals living only in the Andean region of Ecuador, the small sample size may not accurately represent the entire population of Ecuador. As a result, it is unclear whether our findings are generalizable to other regions and Latin American countries. We increased the power of our association study by excluding the effect of the most important confounding variables and by using the Bonferroni correction test. Another limitation is that all healthy staff in the control group were infected during the first wave of the pandemic, at a time when vaccines against SARS-CoV-2 were not available, rather than enrolling individuals in communities. In contrast, the study group was represented by individuals from a single hospital with two doses of vaccine, who required invasive mechanical ventilation and intensive care during the Omicron VOC dominant wave. This certainly introduces a selection bias that may affect the gene distribution. Comparing the results between a group of healthy subjects and a group of individuals with COVID-19 would only help to identify genes associated with susceptibility to the disease, not the clinical spectrum of the phenotype. Given that SARS-CoV-2 has infected most of the world’s population, it may not be feasible to recruit a healthy control group that has not been exposed to SARS-CoV-2. Our goal was to find the gene variants associated with disease severity. Also, because of the low prevalence of each comorbidity reported individually by each patient, it is also likely that a bias could be present. However, we have grouped them together and their combined effects have been calculated in the regression analysis. Based on available data, it appears that the Omicron variant does not have a stronger association with severe COVID-19 than other VoCs. It is likely that hybrid immunity favors a less severe phenotype associated with Omicron VoC. We have taken advantage of this to unmask more clearly how genetic variables affect a complex trait such as COVID-19. A greater influence of genetic factors may be present in previously immunized individuals with the severe form of COVID-19. Thus, we compared individuals who undergone the severe form of the disease and who had already received at least two vaccines from unimmunized subjects with the asymptomatic-mild form from. This design was done to increase the power to detect gene variants related to the severity of the COVID-19 phenotype. The absence of selection bias in our analysis is suggested by HWE and the similarity of allele and genotype frequencies across both groups.

To summarize, while the recessive genetic model showed that *CCL2* rs1024611-G and *OAS1* rs10774671-G gene variants were associated with COVID-19, no association was found for *DPP9* rs10406145 in our study. We could not completely exclude the potential of *DPP9* as a gene associated with the severe COVID-19 phenotype due to undetectable genotype frequencies or small effects of these variants. Genetic association studies of the COVID-19 phenotype are very limited in Latin America, and our research helps to clarify the role of genetic factors in the COVID-19 phenotype. A larger sample size, a replication cohort, and additional SNVs may need to be explored. The experience with COVID-19 provided an opportunity to identify an ethnicity-based approach to recognize genetically high-risk individuals in different populations for emerging diseases. Identifying high-risk individuals who need urgent medical attention is especially important during epidemics. It can be very helpful in formulating policies and allocating resources.

## Data availability statement

The datasets presented in this study can be found in online repositories. The names of the repository/repositories and accession number(s) can be found in the article/**Supplementary Material**.

## Ethics statement

The studies involving humans were approved by The Comite de Etica para la Revisión Expedita de Investigaciones en COVID-19 of the Ministerio de Salud Publica of Ecuador (MSP-CGDES-2020-0244-O1). The studies were conducted in accordance with the local legislation and institutional requirements. The participants provided their written informed consent to participate in this study.

## Author contributions

EC-V: Data curation, Formal analysis, Investigation, Methodology, Software, Validation, Visualization, Writing – original draft. FÁ-N: Conceptualization, Data curation, Formal analysis, Funding acquisition, Investigation, Methodology, Project administration, Resources, Software, Supervision, Validation, Visualization, Writing – original draft, Writing – review & editing. AT-V: Data curation, Formal analysis, Investigation, Methodology, Software, Writing – original draft. TB-D: Writing – original draft. KP: Data curation, Formal analysis, Investigation, Methodology, Software, Writing – original draft. CA-L: Writing – original draft, Formal analysis, Funding acquisition, Investigation, Methodology, Project administration, Supervision, Validation, Writing – review & editing. IP: Conceptualization, Data curation, Investigation, Methodology, Supervision, Writing – original draft. KN: Data curation, Investigation, Methodology, Writing – original draft.
