# Supplementary material for: Single nucleotide variants in the CCL2, OAS1 and DPP9 genes and their association with the severity of COVID-19 in an Ecuadorian population
Source: Front Cell Infect Microbiol. 2024 Apr 17;14:1322882. doi: 10.3389/fcimb.2024.1322882 (PMC11061356; doi:10.3389/fcimb.2024.1322882)
Supplement: Supplementary file 1 [file DataSheet_1.docx]

Supplementary Material

# Supplementary Tables

## Supplementary Table S1

| **Table S1. Information of Three SNVs** | | | | |
| --- | --- | --- | --- | --- |
| Gene Locus | SNV-ID | Chromosome Position (bp) | Localization/Consequence | Base Change |
| *CCL2* (17q12) | rs1024611 | 32579788 | pathogenic, risk-factor | A>G |
| *OAS1* (12q24.13) | rs10774671 | 13357193 | UTR3 | G>A,C |
| *DPPL6* (19p13.3) | rs10406145 | 4692354 | Intron | G>A,C,T |

## Supplementary Table S2

| **Table S2. Primer sequences, annealing temperature, restriction enzymes and fragment sizes of the *CCL2*, *OAS1*, and *DPP9* gene variants.** | | | | | |
| --- | --- | --- | --- | --- | --- |
| **Target sequence** | **Primer sequence** | **Annealing temperature (^0^C)** | **Restriction enzymes** | **PCR product (pb)** | **RFLP fragment (pb)** |
| *CCL2 rs1024611* | F: 5´-gggAgggCATCTTTTCTTgA-3´ | 57 | *Pvu*II | 136 | A: 136 |
|  | R: 5´- AAAgTgACTTggCCTTTgCAT-3´ |  |  |  | G: 91 + 45 |
| *OAS1 rs10774671* | F: 5´- TCC-AgA-Tgg-CAT-gTC-ACA-gT-3´ | 62 | *Alu*I | 306 | G: 306 |
|  | R: 5´- TAg-AAg-gCC-Agg-AgT-CAg-gA-3´ |  |  |  | A: 255 + 51 |
| *DPP9 rs10406145* | F: 5´-TCCTAACCTTTTAggTCTAAACAAggTAC-3´ | 60.5 | *Ban*l | 139 | G: 139 |
|  | R: 5´-gTgggAACCgCTCTCCTC-3´ |  |  |  | C: 114 |
| PCR: polymerase chain reaction; RFLP: restriction fragment length polymorphism. | | | | | |

## Supplementary Table S3

| **Table S3. Components and concentrations of each PCR run** | | | |
| --- | --- | --- | --- |
|  | *CCL2*  rs1024611  (µL) | *OAS1*  rs10774671  (µL) | *DPP9*  rs10406145  (µL) |
| Genomic DNA | 2 | 1.5 | 3 |
| Master Mix |  |  |  |
| Buffer | 3 | 5 | 3 |
| MgCL_2_ | 1.5 | 1 | 1.5 |
| dNTP mix | 0.5 | 0.5 | 0.5 |
| Forward and Reverse Primers | 2 | 1.5 | 1.5 |
| Primer | 2 | 1.5 | 1.5 |
| ddH_2_O | 18.5 | 18.5 | 17.5 |
| DreamTaq Polymerase | 0.5 | 0.5 | 1.5 |
| Total | 30 | 30 | 30 |
|  | | | |

## Supplementary Table S4

| Suplementary Table S4. Tests for Hardy-Weinberg Equilibrium | | | | | | |
| --- | --- | --- | --- | --- | --- | --- |
|  | *CCL2* | | *OAS1* | | *DPP9* | |
| Test | Statistic | *P*-value | Statistic | *P*-value | Statistic | *P*-value |
| Chi-square test: | 0,4384 | 0,5078 | 1,5625 | 0,2113 | 0,7461 | 0,3877 |
| Chi-square test with continuity correction: | 0,2527 | 0,6151 | 0,9804 | 0,3221 | 0,2564 | 0,6126 |
| Likelihood-ratio test: | 0,4366 | 0,5087 | 1,7709 | 0,1833 | 0,67364 | 0,41179 |
| Exact test with selome p-value: | NA | 0,5266 | NA | 0,3471 | NA | 0,39854 |
| Exact test with dost p-value: | NA | 0,6139 | NA | 0,3762 | NA | 0,57563 |
| Exact test with mid p-value: | NA | 0,4614 | NA | 0,2803 | NA | 0,3020 |
| Permutation test: | 0,4384 | 0,5265 | 1,5625 | 0,3508 | 0,74613 | 0,40135 |

## Supplementary Table S5

| **Table S5. Linkage Disequilibrium Test for SNVs of *CCL2*, *OAS1*, and *DPPL6* genes.** | | | |
| --- | --- | --- | --- |
| **SNV** | ***CCL2*** | ***OAS1*** | ***DPPL6*** |
| *CCL2* | ----- | 0.011 | 0.012 |
| *OAS1* | 0.292 | ----- | 0.035 |
| *DPP9* | 0.343 | **0.992** | ----- |
| The lower left value under diagonal indicates the *D´* value and the upper right value on diagonal indicates the *r^2^* value. Bold font represents the stronger LD which was defined with *D'*–value that was closer to 1. | | | |
